# Supplementary material for: External comparators and estimands
Source: Front Drug Saf Regul. 2024 Feb 2;3:1332040. doi: 10.3389/fdsfr.2023.1332040 (PMC12443115; doi:10.3389/fdsfr.2023.1332040)
Supplement: Supplementary file 2 [file DataSheet1.PDF]

External Comparators and Estimands

Gerd Rippin[1]

[1] IQVIA, Biostatistics, Frankfurt a.M., Germany

*** Correspondence:**
Corresponding Author
gerd.rippin@iqvia.com

Keywords: Externally Controlled Trials, External Comparator Cohort Studies, External Comparators, Historical Control Studies, Estimands

Number of words, excluding the abstract, acknowledgements and references: 3019

Number of tables and figures: None

Abstract

The estimand framework as defined by the ICH E9(R1) addendum aims to clearly define "the treatment effect reflecting the clinical question posed by the trial objective" (ICH E9(R1) Expert Working Group, 2021). It intends to achieve this goal by specifying the 5 estimand attributes: population, treatment conditions, endpoints, population-level summary, and handling of intercurrent events (IEs). Hybrid clinical / observational research like External Comparators (ECs) are posing new questions to the estimand framework. They lead to nuanced reflections on existing attributes and considerations for additional ones. This paper reviews estimand attributes in the context of ECs.

1 Introduction

Though there is global agreement that the Randomized Controlled Trial (RCT) is the gold standard for drug approval studies, there are cases where other study designs are needed. External Comparators (ECs) compare an external cohort against a Single-Arm Trial (SAT) (U.S. Food and Drug Administration, 2023, Ghadessi et al., 2020, Burger et al., 2021, Seeger et al., 2020, Skovlund et al., 2018, Thorlund et al., 2020). This mixed clinical / observational research set-up leads to new considerations about the estimand framework as defined by the International Conference of Harmonisation (ICH) E9(R1) addendum (ICH E9(R1) Expert Working Group, 2021). The addendum states that an estimand is the clear definition of "the treatment effect reflecting the clinical question posed by the trial objective". (ICH E9(R1) Expert Working Group, 2021). This goal was suggested to be achieved by specifying 5 estimand attributes: population, treatment conditions, endpoints, population-level summary, and handling of intercurrent events (IEs). General introductions to the estimand framework are available (e.g., Gogtay, 2021), and some specific considerations about observational studies (Li et al., 2022, Wu et al., 2023, Chen et al., 2023) and about EC estimands (Rippin et al., 2022) have been derived as well. This paper builds up on the latter previous work by refining EC estimand approaches.

2 Estimand Attributes

2.1 Estimand Attribute 1: Treatment Conditions

The eligible treatment conditions like doses, route of administration, etc., need to be specified and clearly defined. If different doses or other treatment conditions have different expected treatment effects for the analyzed endpoints, separate analyses must be performed. This is due to the required consistency assumption of causal inference methodology, which involves homogeneity of treatment effects (Faries et al., 2020, Hernán & Robins, 2023).

While comparing the SAT against another single treatment is less complex, comparing against Standard of Care (SoC) can lead to additional difficulties. First, the actual treatments belonging under the umbrella term of the SoC should be characterized thoroughly by means of descriptive statistics, both for best medical understanding and transparency. The definition of SoC can vary by country/region (so stratification of descriptive statistics may be helpful). SoC can also not be as consistent in the RW as it is in a controlled setting. Hence, a critical review of the RW SoC may be necessary to decide whether treatments should be excluded because they are not considered to be appropriate. If the eligible SoC treatments are narrowed down in such a way, some patient groups may no longer be included in the analysis population. Thus, this estimand attribute of treatment conditions may be related to the population attribute. The remaining eligible treatments must also have the same expected treatment effects (again due to the consistency assumption of causal inference). A check for homogeneity of SoC treatment effects is possible and should be performed, at least for the most common treatments with adequate sample size.

Challenges also arise in situations where the treatment exposure (compliance / adherence) is very different between the SAT and the EC. It is possible that the exposure in the SAT's controlled setting is high, while the RW setting may lead to lower treatment exposure. This is due to a strict SAT protocol and strong monitoring of sites and patients to follow the protocol, while there is no such situation in the RW. In such circumstances, it is particularly important to describe treatment exposure in detail by statistical tables. We also need to think about the requirement for any needed minimum exposure or whether applying a hypothetical estimand strategy (see Section 2.4) is a reasonable approach to overcome potentially substantial exposure differences. Requesting a minimal exposure would introduce immortal time bias, which needs to be handled statistically in a correct manner.

Special challenges may arise if the trial has (or needs to be compared versus) a dynamic treatment strategy. For example, when the trial's design (or the comparator) includes dose escalation, the statistical handling of post-baseline dosing events may involve complex analytical approaches in line with deriving hypothetical estimands as per the estimand attribute 4 (Section 2.4).

2.2 Estimand Attribute 2: Population

2.2.1 Intention-to-Treat (ITT) Population Versus Safety Population

As a general principle, there is a preference to compare populations which share a common definition to compare like-for-like (Pocock, 1976, Gray et al., 2022). However, the EC is typically compiled from real word (RW) data sources, where no Intention-to-Treat (ITT) population exists, which denotes the population which was intended to be treated (for example at enrollment or randomization). This may lead to leveraging for both cohorts rather the population which received the treatment (the Safety population), which has the advantage of straightforward results

This is a provisional file, not the final typeset article

interpretation but the disadvantage of applying an approach which is outside common RCT standards.

As an alternative, it is possible to argue that taking the trial's ITT population and the external Safety population leads to a conservative analysis, because the treatment effect should be lower in the population which is just intended to be treated compared to the population which actually has been treated. The advantage is that such an approach is in line with the trial analysis which typically relies on the ITT population. The disadvantage is that the comparison is only unbiased if there is no difference for the EC in terms of the ITT and Safety population. Also, by taking the trial's ITT population and the external Safety population, the intentionally induced bias (conservativeness) is potentially beyond a reasonable margin.

A practical example for considering either approach could be the chimeric antigen receptor (CAR) – T cell treatment where there are usually several weeks after baseline (index date, time zero) until the treatment is received, which could mean that there is a meaningful drop-out after baseline but before the treatment is taken. Considering this drop-out or not by means of choosing the ITT or Safety analysis population leads to a likely difference in the estimated treatment effect. Specifically, the treatment effect based on the ITT population is expected to be lower than the one based on the Safety population. Specifying more than one estimand (one as the primary estimand and the other as a supplementary analysis) could be helpful to generate multiple perspectives.

2.2.2 Approximation of the Target Population

Although the trial's eligibility criteria are mimicked in the EC as much as possible, some baseline information is likely to not be available in the RW. For example, an HIV test result or an ECOG value is not always measured or documented in RW datasets. Hence, the RW analysis population is in most cases (if not in all) an approximation of the population of interest. However, if the SAT's baseline covariates' values are already available, there is an additional possibility to refine populations. Instead of just applying the trial's eligibility criteria to the EC as much as possible, it can also be considered to restrict the covariate value ranges according to what has been observed in the trial. If, for example, the eligibility criteria require patients to be at least 18 years old, there is the possibility to look up the actual trial's age range, which hypothetically may be between 43 and 82 years. Then, the EC could be restricted to the same age range and this approach could be applied for all other important covariates as well. Although this seems to make sense statistically and is in line with the concept of exchangeability (Pocock, 1976, Gray et al., 2020), there is no guidance yet whether such an approach would be considered preferable from a regulatory or payers' perspective.

Another approximation regarding the included population occurs due to eligibility values being typically taken from a time window (look-back period) before the EC baseline (e.g., 3, 6 or 12 months before true baseline). This is often needed because the RW measurements for important eligibility criteria are not necessarily available at the exact time of baseline. However, the longer the eligibility measurements are retrieved from the past, the more likely the occurrence of measurement error, which can either be pure random error or a systematic shift; for example, for an indication where the patient's condition is deteriorating fast. Both types of errors would lead to issues, but a systematic shift could directly lead to the analysis becoming anti-conservative.

Often, there is a trade-off between the quality of the target population approximation in the EC and sample size, for example in case of baseline data being substantially missing. One approach is to derive not a single but two populations: one being a narrow data subset approximating the target population to the highest possible degree, and one being a broad subset of the data with a less

accurate population approximation but higher sample size. Presenting results based on more than one population may be helpful to check for the robustness of the generated evidence.

The comparator population can be derived either by a retrospective approach or by a new prospective cohort study. The retrospective approach has the advantage to generate results quicker, which can be an important aspect, while the prospective approach comes with the advantage of fully customized data collection, which leads most probably to a better target population approximation.

2.3 Estimand Attribute 3: Endpoints

While endpoints like Overall Survival (OS) are typically comparable across cohorts (Rippin et al, 2022), others like progression-free survival (PFS) or overall response rate (ORR) may be less comparable. This is due to progression being typically not measured in the RW by internationally standardized classification rules like Response Evaluation Criteria in Solid Tumours (RECIST) (Eisenhauer et al., 2009) or Lugano classification (Cheson et al., 2014). Thus, comparisons of response-based endpoints in the context of ECs require extra caution to handle potential issues of endpoint misclassification, for example by statistical approaches involving internal or external validation (e.g., central and blinded independent review of endpoint data).

One other source of non-comparability may occur for composite time-to-event (TTE) endpoints, such as PFS, Time to Next Treatment or Death, or Duration of Response until Progression or Death. These endpoints are not automatically comparable across different data sources due to potentially different censoring proportions for the earlier part of the composite endpoint. As an example, consider PFS with high missingness for progression but low missingness for survival. In such a case, PFS is shifted towards the endpoint survival. Because there is typically more missingness in RW data sources, composite TTE endpoints can pose specific issues for ECs.

The endpoint specification is also required for a clear definition of what constitutes an acceptable measurement timepoint, since bias may occur when endpoints are measured at different times across cohorts. One option is to define time windows around a specific target time (e.g., 3 months after baseline +/- 2 weeks). For repeated measurements analyses (e.g., for patient reported outcomes) there are further options to handle a differential frequency of endpoint measurements across cohorts by applying additional statistical assumptions modelling the endpoint over time.

For TTE endpoints, different measurement times can be handled accurately by interval-censoring methods (Bogaerts et al., 2017), which would also handle the case when more frequent follow-up visits occur for more severe cases (intensity bias).

Safety outcomes are typically not as exhaustively documented in the RW as in trials, therefore these endpoints are typically not comparable. However, there may be a complete list of major / life-threatening events or hospitalizations / doctor's visits in some RW data sources, which may enable comparisons of key safety events.

2.4 Estimand Attribute 4: Handling of Intercurrent Events

The ICH E9 (R1) guideline defines IEs as "Events occurring after treatment initiation that affect either the interpretation or the existence of the measurements associated with the clinical question of interest." (ICH E9(R1) addendum, 2021), and outlines 5 strategies on how to handle IEs. Although a full discussion of all IE handling strategies is out of scope of this paper, the *treatment policy strategy* and the *hypothetical estimand strategy* will be highlighted briefly. For the former strategy "the

This is a provisional file, not the final typeset article

intercurrent event is considered irrelevant in defining the treatment effect of interest” (ICH E9(R1) addendum, 2021), while the latter is setting up a hypothetical scenario where the IE would not have occurred.

The question of which IE handling strategy is best suited for ECs is especially important to clarify because IEs can differ more in EC studies compared to RCTs due to different settings and data temporality across cohorts. For example, the kind and timing of subsequent treatments may be completely different in the EC. In such cases, the differential effect of follow-up therapies may bias comparisons considerably, especially for long-term endpoints like OS where the effects of subsequent treatments may be substantial. In this case, alternatives to the treatment policy estimand may be important to consider, for example, by applying the hypothetical estimand strategy. For short-term endpoints, IEs may occur less often, leading to a stronger rationale for the treatment policy estimand being the primary estimand. It may also be helpful to define more than one estimand for a robust description of study results.

2.5 Estimand Attribute 5: Population-level summary

Some population-level summaries may also be more appropriate than others for ECs. This is due to the fact that some summaries need assumptions which can become fragile in ECs. As an example, it was seen before that the proportional hazards assumption is easily lost when comparing data across different data sources (Hoogendoorn et al., 2022). This leads to issues when using the Cox model and selecting the hazard ratio as the population-level summary. Note that the Cox model was criticized since the last decade also for other reasons (Aalen et al., 2015, Hernán, 2010, Mao et al., 2018, Rufibach et al., 2019, Martinussen et al., 2020, Stensrud et al., 2019). Accelerated Failure Time (AFT) models (Collett, 2023) may also not be sufficiently flexible to model the data successfully due to the selected parametric approach. Thus, alternative TTE population-level summaries are recommended, e.g., restricted mean survival time (RMST) differences (Collett, 2023).

Other issues with population-level summaries in an EC setting may generally occur due to the induced heterogeneity across cohorts. For example, the variance across cohorts is more likely to be heteroscedastic compared to an RCT setting, for example for a repeated measurements model analyzing quality-of-life questionnaires. Hence, statistical models without strong assumptions should be preferred, and for the described example above allowing for unequal variances across cohorts is recommended.

2.6 Further Considerations

The ICH E9(R1) addendum focusses on clinical research. However, ECs are outside of the clinical trial setting, such that additional considerations are needed. One is the definition of baseline and another one is the specification of the marginal estimator, which are discussed below.

2.6.1 The Definition of Baseline

It is not always clear how baseline should be defined for ECs (see also Section 2.2). One example is when the treatment is a combination of two or more drugs. One possibility to define baseline is to take the latest start date of the involved treatments. A decision should however only be reached after having receiving input from medical team members. If one element of the combination treatment is the major driver for efficacy / effectiveness or one part of the combination treatment relates to controlling adverse events only, there may be rationale to choose baseline to be the corresponding treatment element start date. Another possibility may be to restrict the population which starts taking

all treatment elements either at the same time or within a certain time window (e.g., ± 1 week), especially if there are treatment guidelines stating that all components of the treatment should be taken at the same time.

Further, there may be situations where a comparison needs to take place against comparator treatments which are unknown to be effective. In such cases, it is questionable whether any treatment start date in the comparator cohort would constitute the best baseline definition, since the treatment start may be an irrelevant event for disease progression. Also, its timing may be a long time after disease progression, because it was not considered critical that the treatment was prescribed or taken.

All of these considerations do show that the comparator's effectiveness does matter when deciding about the baseline definition. If the comparator drug is effective, it is natural to take the treatment start date as baseline across both cohorts, but if not effective it remains to be checked for the specific study at hand whether such an event date is meaningful to define baseline.

Moreover, there are cases where no treatment is available at all (Wakabayashi et al., 2023), such that it is simply not possible to define baseline by means of any treatment start date.

As an alternative baseline, it may be possible for some indications to use the last disease progression or another important clinical event (myocardial infarct, stroke, etc.). However, survivor bias is introduced for the trial participants because the time from the clinical event to the treatment start date has been survived in the trial population, and methods to handle survivor bias (Wang, 2022) need to be applied for the statistical analysis of the data.

The longer the index date in the past (relative to the treatment start date), the more pronounced the disadvantages. Firstly, the immortal time bias may become critically large, and secondly, potentially unnecessary variability in terms of "white noise" is introduced to the analysis. Note that if the treatment start date is chosen to be baseline, time from a clinical event to treatment start may be used as a covariate in causal inference approaches.

Moreover, there are cases where patients are eligible multiple times, for example at the start date of the third and higher lines of treatment (Backenroth, 2021, Hatswell et al., 2022), and using all of the available data by allowing patients to enter the study at multiple timepoints (multiple baselines) may be an efficient solution, though more complex statistical methodology is needed to handle correlated data.

Of note, when discussing best baseline definitions, issues may also arise about the timing of the covariate measurements. Firstly, covariates measured at the original SAT baseline are becoming post-baseline covariates if the index date is moved to an earlier time point like disease progression. This is problematic from a statistical theory perspective, as only baseline variables can be used in standard statistical approaches for covariate adjustment. Secondly, the EC typically applies a look-back period to derive eligible baseline measurements (see Section 2.2.2). This may lead to actual covariate values at true baseline being potentially different from previously recorded values, which is a threat to the validity of causal inference methods.

Of course, the definition of baseline is also related to the population and treatment attributes, but handling this topic separately is considered to be useful, for example, by helping to identify survivor bias and to clarify whether or not patients will be allowed to enter the study at multiple baseline times.

This is a provisional file, not the final typeset article

2.6.2 The Marginal Estimator

While "RCTs are designed to provide estimates of the average treatment effect" (van Amsterdam and Ranganath, 2023), ECs may estimate the Average Treatment Effect (ATE), the Average Treatment Effect on the Treated (ATT), the Average Treatment Effect on the Untreated (ATU) or the Average Treatment Effect in the Overlap Population (ATO) (Rippin et al., 2022). While each of these marginal estimators come with their own advantages and disadvantages (Rippin et al., 2022), none of them are yet directly included in the ICH estimand framework. Although the framework speaks about estimators, the estimators are supposed to have an effect on the estimate only in terms of how well model assumptions are met. A conceptual change of the estimate is a conceptual change in the estimand and cannot be attributed to the estimator level. Hence, the natural idea to classify the marginal estimator to the estimator level does fail.

One idea to integrate marginal estimators into the framework is to use a sub-attribute to the population-level summary attribute, for example, by saying that the ATE hazard ratio or the ATT relative risk is estimated. However, the population-level summary (e.g., a relative risk) is specified independently from the marginal estimator, such that this would constitute a mix of two independent concepts.

3 Discussion

The ICH E9(R1) focuses on clinical trials but states that its principles are relevant for observational studies as well (ICH E9(R1) Expert Working Group, 2021). However, setting up an EC estimand is less straightforward than in clinical trials, which is in line with Wu et al., 2023 stating that "constructing estimands for real-world evidence (RWE) studies might require additional considerations". These additional considerations have been presented for EC studies in this paper. It was seen that due to specific challenges which are typically not seen in clinical trials, nuanced reflections are needed for the 5 existing estimand attributes. Moreover, there are further important aspects which need to be addressed in the EC setting.

The first is the definition of baseline, and the second is the specification of the marginal estimator. The Li paper (Li et al., 2022) suggested that there is a relationship of the marginal estimator with the population attribute, but this seems to be against the spirit of the ICH E9(R1) addendum, which treats the population attribute in the sense of defining eligibility (population selection), such that we suggest rather a separate attribute or a specification as per the population-level summary.

In case the estimand framework is revised to include RW study needs, the baseline definition and the marginal estimator are candidates for additional estimand attributes. Further new attributes may be the quality and completeness of data and covariates because valid causal inference depends on data being fit for purpose, as mentioned by Chen et al., 2023, who have been discussing estimands in the broader context of RW studies.

We hope that this paper not only fosters discussions around the set-up of EC estimands but also about the estimand framework in general.

4 Conflict of Interest

None.

5 Funding

This article was not funded.

6 References

1. U.S. Food and Drug Administration (2023). Considerations for the design and conduct of externally controlled trials for drug and biological products. Draft Guidance for Industry. https://www.fda.gov/regulatory-information/search-fda-guidance-documents/considerations-design-and-conduct-externally-controlled-trials-drug-and-biological-products. (Accessed October 8, 2023.)
2. Ghadessi, M., Tang, R., Zhou, J. et al. (2020). A roadmap to using historical controls in clinical trials – by Drug Information Association Adaptive Design Scientific Working Group (DIA-ADSWG). *Orphanet J Rare Dis.* 15 (1), 1-19. http://dx.doi.org/10.1186/s13023-020-1332-x.
3. Burger, H.U., Gerlinger, C., Harbron, C. et al. (2021). The use of external controls: To what extent can it currently be recommended? *Pharm Stat* 20 (6), 1002-1016. http://dx.doi.org/10.1002/pst.2120.
4. Seeger, J.D., Davis, K.J., Innacone, M.R. et al. (2020). Methods for external control groups for single arm trials or long-term uncontrolled extensions to randomized clinical trials. Pharmacoepidemiol *Drug Saf.* 29, 1382-1392. http://dx.doi.org/10.1002/pds.5141.
5. Skovlund, E., Leufkens, H.G.M., Smyth, J.F. (2018). The use of real-world data in cancer drug development. *Eur J Cancer* 101, 69-76. http://dx.doi.org/10.1016/j.ejca.2018.06.036.
6. Thorlund, K., Dron, L., Park, J.J.H., et al. (2020). Synthetic and external controls in clinical trials – a primer for researchers. *Clin Epi* 12, 457-467. http://doi.org/10.2147/CLEP.S242097.
7. ICH E9(R1) Expert Working Group (2021). ICH E9(R1) addendum on estimands and sensitivity analysis in clinical trials to the guideline on statistical principles for clinical trials. EMA/CHMP/ICH/436221/2017. https://www.fda.gov/media/148473/download. (Accessed Oct 8, 2023.)
8. Gogtay, N.J., Ranganathan, P., Aggarwal, R. (2021). Understanding estimands. *Perspect Clin Res* 12 (2), 106-112. http://dx.doi.org/10.4103/picr.picr_384_20.
9. Li, H. Wang, C., Chen, W-C. (2022) Estimand in observational studies: some considerations beyond ICH E9 (R1). *Pharm Stat.* 21 (5), 835-844. http://dx.doi.org/10.1002/pst.2196.
10. Wu, Y., Wang, H., Chen, J., et al. (2023). Estimand in Real-World Evidence Study: From Frameworks to Application. In: He, W., Fang, Y., Wang, H. (eds) Real-World Evidence in Medical Product Development . Springer, Cham. https://doi.org/10.1007/978-3-031-26328-6_9.
11. Chen, J., Scharfstein , D., Wang, H. et al. (2023). Estimands in real-world evidence studies. *Stat in Biopharm Res*. (online pre-print) https://doi.org/10.1080/19466315.2023.2259829.
12. Rippin, G., Ballarini, N., Sanz, H., Largent, J., Quinten, C., Pignatti, F. (2022). A review of causal inference for external comparator arm studies. *Drug Saf* 45 (8), 815-837. https://doi.org/10.1007/s40264-022-01206-y.
13. Pocock, S. (1976). The combination of randomized and historical controls in clinical trials. *J Chronic Dis.* 29, 175–88. https://doi.org/10.1016/0021-9681(76)90044-8.
14. Gray, C.M., Grimson, F., Layton. D. et al. (2020). A framework for methodological choice and evidence assessment for studies using external comparators from real-world data. *Drug Saf* 43, 623–33. https://doi.org/10.1007/s40264-020-00944-1.
15. Faries, D.E., Zhang, X., Zbigniew, K., et al. (2020). Real world health care data analysis using SAS: causal methods and implementation using SAS. SAS Institute Inc., Cary, NC.
16. Hernán, M.A., Robins, J.M. (2023). Causal inference: What if. Chapman & Hall/CRC, Boca Raton, FL. https://doi.org/10.1201/9781315374932.

This is a provisional file, not the final typeset article

17. Eisenhauer, E.A., Therasse, P., Bogaerts, J., et al. (2009). New response evaluation criteria in solid tumours: revised RECIST guideline (version 1.1). *Eur J Cancer* 45, 228-247. http://dx.doi.org/10.1016/j.ejca.2008.10.026.
18. Cheson, B.D., Fisher, R.I., Barrington, S.F., et al. (2014). Recommendations for initial evaluation, staging, and response assessment of Hodgkin and non-Hodgkin lymphoma: the Lugano classification *J Clin Onc* 32 (27), 3059-3067.
19. Bogaerts, K., Komarek, A., Lesaffre, E. (2017). Survival analysis with interval-censored data. A practical approach with examples in R, SAS and BUGS. Chapman & Hall/CRC, New York. http://dx.doi.org/10.1201/9781315116945.
20. Hoogendoorn, W.E., Sanz, H., Quinten, C. et al. (2022). A methodology study to evaluate ECA study results versus RCT results: Multiple Myeloma case study. Poster at 38th ICPE, Copenhagen, Denmark. https://pharmacoepi.org/pub/?id=A15839AD-B6E4-D28E-1C20-FA5A5B1D2B50**.** (Accessed Oct 8, 2023.)
21. Aalen, O.O., Cook, R.J., Roysland, K. (2015). Does Cox analysis of a randomized survival study yield a causal treatment effect? *Lifetime Data Anal* 21 (4), 579-593. http://dx.doi.org/10.1007/s10985-015-9335-y**.**
22. Hernán, M.A. (2010). The hazards of hazard ratios. *Epidem* 21 (1), 13-15. http://dx.doi.org/10.1097/EDE.0b013e3181c1ea43.
23. Mao, H., Li, L., Yang. W. et al. (2018). On the propensity score weighting analysis with survival outcome: Estimands, estimation, and inference. *Stat Med* 37 (26), 3745-3763. http://dx.doi.org/10.1002/sim.7839.
24. Rufibach, K. (2019). Treatment effect quantification for time-to-event endpoints – estimands, analysis strategies, and beyond. *Pharm Stat* 18 (2), 145-165. http://dx.doi.org/10.1002/pst.1917.
25. Martinussen, T., Vansteelandt, S., Andersen, P.K. (2020). Subtleties in the interpretation of hazard contrasts. *Lifetime Data Anal* 26 (4), 833-855. http://dx.doi.org/10.1007/s10985-020-09501-5.
26. Stensrud, M.J., Aalen, J.M., Aalen, O.O. et al. (2019). Limitations of hazard ratios in clinical trials. *Eur Heart J* 40, 1378-1383. http://dx.doi.org/10.1093/eurheartj/ehy770.
27. Collett D. Modelling survival data in medical research. Fourth edition, 2023. Chapman & Hall/CRC, New York. https://doi.org/10.1201/9781003282525.
28. Wakabayashi, R., Hirano, T., Laurent, T., et al. (2023). Impact of ‘time zero’ of follow-up settings in a comparative effectiveness study using real-world data with a non-user comparator: Comparison of Six Different Settings. *Drugs - Real World Outc* 10, 107–17. http://dx.doi.org/10.1007/s40801-022-00343-1.
29. Wang, J., Peduzzi, P., Wininger, M. et al. (2022). Statistical methods for accommodating immortal time: a selective review and comparison. https://arxiv.org/abs/2202.02369. (Accessed Oct 8, 2023.)
30. Backenroth, D. (2021). How to choose a time zero for patients in external control arms. *Pharm Stat* 20, 783–92. http://dx.doi.org/10.1002/pst.2107.
31. Hatswell, A.J., Deighton, K., Snider, J.T. et al. (2022). Approaches to selecting ‘‘time zero’’ in external control arms with multiple potential entry points: a simulation study of 8 approaches. *Medic Dec Making* 42 (7), 893–905. http://dx.doi.org/10.1177/0272989X221096070.
32. van Amsterdam, W.A.C. and Ranganath, R. (2023). Conditional average treatment effect estimation with marginally constrained models. *J Caus Inf* 11 (1), pp. 20220027. https://doi.org/10.1515/jci-2022-0027.

7 Data Availability Statement

Not applicable.

Table 1: Estimand Attributes and Further Considerations

Attributes & Further Considerations	Issues and Pitfalls	Opportunities
Attribute 1: Treatment conditions	• Issues /complexity when comparing against SoC<br>• Differential exposure across cohorts<br>• Minimum exposure required?<br>•	• Perform a thorough check that statistical assumptions for SoC comparisons are holding<br>• If needed, apply statistical solutions to adjust for differential exposure
Attribute 2: Population	• ITT or Safety population?<br>• Data availability<br>• Data quality (measurement error)<br>• Inappropriate look-back period to derive eligibility	• Definition of a “broad” and “narrow” population<br>• Harmonization of covariate ranges
Attribute 3: Endpoints	• Differential measurement methods or timings of endpoints<br>• Misclassification	• Definition of time windows for eligible measurements<br>• Advanced statistical handling of differences in measurement methods and timings<br>• Independent blinded review of endpoints<br>• Other internal or external validation approaches
Attribute 4: Handling of Intercurrent Events (IEs)	• IEs may be very different across cohorts	• Apply statistical adjustment to handle the effects of IEs correctly<br>• Specify more than one strategy to handle IEs
Attribute 5: Population-level summary	• Some statistical assumptions more fragile when data is joined from different data sources	• Prefer statistical approaches with mild assumptions over ones with strong assumptions
Further Consideration 1: Baseline	• Baseline definition in case of combination treatments, ineffective treatments or non-treatment patients<br>• Alignment issues of baseline and covariate measurement timings	• Check alternative baseline definitions, e.g., according to a recent major clinical event<br>• Apply more than one baseline definition

This is a provisional file, not the final typeset article

		• Patients being eligible at multiple times may be included several times by means of multiple baselines
Further Consideration 2: Marginal Estimator	• Best choice of marginal estimator may not be clear	• Opportunity to derive results according to more than one marginal estimator

SoC: Standard of Care. ITT: Intention-to-treat.
